# Polypharmacy and drug interactions in the management of rheumatoid arthritis

**DOI:** 10.1590/1806-9282.20250151

**Published:** 2025-08-08

**Authors:** Lorena Batista Boeing, Natalie Sbalqueiro Fogaça, Barbara Stadler Kahlow, Thelma Skare, Renato Nisihara

**Affiliations:** 1 Mackenzie Evangelical School of Medicine – Curitiba (PR), Brazil.

**Keywords:** Rheumatoid arthritis, Polypharmacy, Drug interactions

## Abstract

**BACKGROUND:**

Rheumatoid arthritis is a chronic inflammatory disease that often necessitates combination therapy. The presence of comorbidities, frequently observed in rheumatoid arthritis patients, can further increase the number of medications required, raising the risk of drug-to-drug interactions.

**OBJECTIVE:**

This study aimed to analyze the prevalence of polypharmacy and identify potential drug-to-drug interactions in rheumatoid arthritis patients using the MedScape® digital platform.

**METHODS:**

A retrospective analysis was conducted on 370 rheumatoid arthritis patients. Data on epidemiological characteristics, comorbidities, and the number of medications used during treatment were collected. The MedScape® platform was employed to assess potential drug-to-drug interactions.

**RESULTS:**

Polypharmacy was observed in 81.1% of the patients, with a higher prevalence among older individuals and those with multiple comorbidities. The MedScape® platform identified 2,018 potential drug-to-drug interactions in the sample, of which 27.5% were mild, 54.4% moderate, and 17.8% severe. Severe interactions frequently involved combinations of antirheumatic drugs.

**CONCLUSIONS:**

Polypharmacy is highly prevalent in rheumatoid arthritis treatment, particularly in patients with comorbidities, increasing the risk of clinically significant drug-to-drug interactions. Physicians should remain vigilant in monitoring for potential interactions and adopt strategies to mitigate the risks, such as regular medication reviews and utilizing digital platforms to support clinical decision-making. This approach is critical to optimizing patient safety and treatment outcomes in rheumatoid arthritis care.

## INTRODUCTION

Polypharmacy is becoming more common in medical practice and is generally defined as the concurrent use of five or more medications by an individual, although there is no consensus on its exact definition^1,2^. It has been shown that polypharmacy is associated with factors such as advancing age, access to ­medications, and the presence of comorbidities^3,4^. Polypharmacy can lead to harmful drug interactions, increasing morbidity, mortality, and care complexity, while also creating a significant financial burden on both patients and the healthcare system^4^.

Rheumatoid arthritis (RA) is a chronic inflammatory ­disease with evolving treatment options, often involving combination therapy. The goal is to reduce or eliminate inflammation, ­minimizing joint damage and cardiovascular complications, which often requires the use of multiple medications^5^. Moreover, RA predominantly affects older individuals with multiple comorbidities, which explains its high prevalence in this population^6^.

A study of 1,101 RA patients found an average of five medications per patient. Those using more than 10 drugs faced higher hospitalization risks due to comorbidities, infections, and adverse drug reactions^7^.

The objective of this study was to assess polypharmacy and drug interactions in a sample of Brazilian patients with RA, given the limited research available in this area. The assessment of drug interactions was conducted using the calculator provided by the MedScape^®^ digital platform.

## METHODS

This is a retrospective study approved by the Institutional Research Ethics Committee under protocol 5455137.

This is a retrospective analytical study based on electronic medical records of patients diagnosed with RA who were undergoing treatment at an outpatient clinic of the Public Health System. Data were collected from patients undergoing treatment between January 2021 and March 2022. Inclusion criteria were patients over 18 who met at least six points of the 2010 American College of Rheumatology/European Alliance of Associations for Rheumatology (ACR/EULAR) Classification Criteria for RA^8^.

Medical records were accessed through the electronic system of Hospital Universitário Evangélico Mackenzie, allowing the retrieval of RA patient records. Patients who died during follow-up or had incomplete data were still included in the analysis. Data collection included:

Epidemiological and clinical data: Age, sex, disease duration, comorbidities, and long-term use of medications. For the purposes of this study, polypharmacy was defined as the concurrent and continuous use of five or more medications for a period of at least 30 days, as documented in the patient's ­medical prescription at the time of the clinical evaluation^2^.

Study of drug interactions: All medications prescribed to each patient were checked for interactions using the MedScape^®^ platform (https://reference.medscape.com/druginteractionchecker), which evaluates interaction presence, severity, potential mechanisms, and suggests therapy adjustments. Interactions were classified as mild, moderate (requiring close monitoring), or serious (possibly needing alternative treatments). The platform can detect interactions in up to 30 drugs, herbs, or foods at once. It was chosen due to its free online access and user-friendly interface, offering unlimited drug interaction calculations, along with additional features like articles and medication-related functions^9^.

### Statistical analysis

Continuous data were expressed in mean and standard deviation for parametric data and median and interquartile range (IQR) for non-parametric data and compared using an unpaired t-test or Mann-Whitney test. Categorical data were expressed in percentages and compared using chi-squared and Fisher tests. Tests were performed using the GraphPad Prism version 9.5.1 software for Windows, GraphPad Software, San Diego, California, USA, www.graphpad.com. The adopted significance level was 5%.

## RESULTS

Data were collected from 370 patients with RA, of whom 87.9% were female. The mean age was 60.1±10.6 years, and the median disease duration was 13 years (IQR: 9–19).

Table 1 summarizes the primary comorbidities observed in the sample and the medications commonly used. Only 8.9% of patients had no comorbidities, with a mean of 2.9 comorbidities per patient. Regarding pharmacological treatment, 81.1% of patients were on five or more medications, ­indicating polypharmacy. Specifically, 8.7% used up to three drugs, 10.1% used four drugs, 53.7% used between five and ten drugs, and 27.2% were on more than ten drugs. Table 2 compares patients using up to five drugs with those using five or more. The analysis revealed that older age and a higher number of ­comorbidities were significantly associated with polypharmacy.

**Table 1:** Main comorbidities and drugs frequently used in the treatment of patients with rheumatoid arthritis (n=370).

Comorbidities		
	Arterial hypertension	40.9% (n=157)
	Osteoporosis/osteopenia	34.1% (n=113)
	Dyslipidemia	29.4% (n=106)
	Osteoarthritis	27.6% (n=87)
	Hypothyroidism	22.7% (n=74)
	Fibromyalgia	19.3% (n=70)
	Diabetes	15.6% (n=60)
Drugs used		
	Glucocorticoid	51.2% (n=189)
	Vitamin D	50.4% (n=186)
	Leflunomide	49.3% (n=182)
	Calcium	48.0% (n=177)
	Methotrexate	43.7% (n=161)
	Folic acid	43.7% (n=161)
	Simvastatin	37.6% (n=139)
	Omeprazole	37.0% (n=136)
	Losartan	29.3% (n=108)
	Paracetamol	26.1% (n=96)
	Levothyroxine	22.9% (n=84)
	Fluoxetine	20.0% (n=74)
	Hydrochlorothiazide	17.0% (n=62)
	Amitriptyline	15.2% (n=56)
	Alendronate	14.6% (n=54)
	Metformin	13.8% (n=51)
	Enalapril	13.0% (n=48)
	Dipyrone	12.8% (n=47)
	Antimalarials	12.0% (n=44)
	Gabapentin	11.7% (n=43)
	AAS	10.9% (n=40)
	Anti-TNF-α	8.6% (n=31)
	Rituximab	4.3% (n=15)
	Anti-interleukin-6	2.7% (n=9)
	Jak inhibitors	2.4% (n=8)

AAS: acetylsalicylic acid.

**Table 2:** Epidemiological data comparison between rheumatoid arthritis patients who met the polypharmacy criteria and those who did not meet the polypharmacy criteria.

	Did not meet polypharmacy criteria n=70	Met polypharmacy criteria n=300	p
Sex: female/male—n	59/11	267/33	0.27
Mean age—years±SD	55.4±11.6	61.3±10.1	<0.0001
Median disease duration—years (IQR)	11.0 (7.0–19.0)	13.0 (9.0–19.0)	0.10
Median number of comorbidities (IQR)	1 (0.0–2.0)	3.0 (2.0–4.0)	<0.0001

SD: standard deviation; n: number; IQR: interquartile range.

A total of 2,018 drug interactions were identified using the Medscape^®^ platform. The distribution of drug interactions showed that 12.5% of patients had no interactions, 31.2% had two interactions, 16.8% had four, 9.8% had six, 9.0% had eight, and 20.7% experienced ten or more interactions. Of these interactions, 27.5% were classified as mild, 54.4% as moderate, and 17.8% as severe.

Table 3 details the most severe drug interactions identified by the platform and provides explanations for their classifications.

**Table 3:** Main serious interactions found in prescriptions for the treatment of rheumatoid arthritis patients (n=2,018).

Drugs	Frequency	Interaction
Prednisone+simvastatin	19.5%	Prednisone reduces simvastatin levels through CYP3A4 metabolism.
Leflunomide+methotrexate	17.2%	Leflunomide increases methotrexate toxicity (hepatotoxicity, pancytopenia) by pharmacodynamic synergism.
Amitriptyline+fluoxetine	9.6%	Fluoxetine and amitriptyline increase serotonin levels.
AAS+methotrexate	5.2%	AAS increases methotrexate levels, decreasing the renal clearance. The risk is higher when methotrexate is used in high doses.
Amlodipine+simvastatin	4.9%	Amlodipine increases simvastatin levels with a potential increase in the risk of myopathy/rhabdomyolysis.
Adalimumab+simvastatin	3.2%	Adalimumab and leflunomide increase immunosuppression with increased infection risk.
AAs+enalapril	3.2%	Aspirin and enalapril had pharmacodynamic antagonism. Non-steroidal anti-inflammatory drugs reduce the anti-hypertensive action of angiotensin-converting enzyme inhibitors.
Antimalarial+leflunomide	2.6%	Increase immunosuppression; risk of infection.
Duloxetine+omeprazole	2.0%	Omeprazole reduces duloxetine's action (metabolism by CYP1A2).
Etanercept+leflunomide	2.0%	Increase immunosuppression; risk of infection.
Leflunomide+tocilizumab	1.7%	Increase immunosuppression; risk of infection.
Prednisone+tofacitinib	1.4%	Increase immunosuppression; risk of infection.
Fluoxetine+antimalarial	1.4%	Both may increase the QTc interval.

AAS: acetylsalicylic acid.

## DISCUSSION

The findings of this study show that polypharmacy is highly prevalent among RA patients, particularly older adults with comorbidities. This observation is consistent with a recent study by Lozano-Lozano et al., which identified hypertension, osteoarthritis, and thyroid disorders as the comorbidities most associated with drug interactions—findings that align with our own^10^. The MedScape platform identified a higher risk of drug interactions in these patients. It is widely recognized as a comprehensive tool for geriatric and pediatric care, covering over 80,000 drugs and available for free^11,12^.

Regarding the epidemiology of polypharmacy, previous studies have found that females had a higher prevalence of polypharmacy compared to males^7,10^. However, in the present study, males were underrepresented, which may explain the disparity; RA is a disease with a higher prevalence in females.

The MedScape platform recommends using the interactions table for serious interactions, which should be approached with caution. The table lists immunosuppressive drugs that increase infection risk, such as adalimumab, leflunomide, etanercept, prednisone, and tofacitinib, commonly used in rheumatology to manage autoimmune diseases. According to EULAR recommendations, combining synthetic disease-modifying antirheumatic drugs (DMARDs) with biotherapy is the standard treatment for better long-term clinical and radiographic outcomes^11,12^. Moreover, the combination of a synthetic immunosuppressor drug such as methotrexate or leflunomide with biologic drugs has the advantage of reducing the immunogenicity against the biologic drugs, extending their efficacy^13^. However, it is essential to consider the increased risk of infection by using this combination, which may be evident to the rheumatologist, but not always to the general practitioner. In addition, RA patients in this study have several comorbidities that may require treatment from specialists in other medical fields, who may not be familiar with rheumatological medications.

An interaction that has been considered serious by the MedScape platform is that between methotrexate and leflunomide. Both drugs have the potential to cause liver damage and may be toxic to the bone marrow, requiring additional care when used in combination^14-16^. This combination was also observed with a prevalence of 46.5% in a study by Lozano-Lozano et al., which used the BOT Plus Pharmacological Surveillance System^10^. Most guidelines recommend using these drugs sequentially, but in developing countries with economic challenges, this combination is considered a viable alternative to biological drugs. A multicenter registry-based study (BiobadaBrasil) of 452 patients using methotrexate and leflunomide showed a favorable safety profile^17^. This combination was also studied in the treatment of psoriatic arthritis in a randomized control trial with 78 participants, and it was shown to be effective, although less well-tolerated from a gastrointestinal perspective compared to methotrexate alone^18,19^.

Among the 361 significant drug interactions identified, the most prevalent was between prednisone and simvastatin. Both medications are known to exert deleterious effects on skeletal muscle, a concern particularly pertinent for patients with RA, who may already experience compromised muscle function due to inflammation-induced sarcopenia and reduced mobility^6^. Prednisone remains a cornerstone in RA management, while simvastatin is frequently prescribed to address the elevated prevalence of arterial hypertension and cardiovascular disease within this patient population. The widespread prescription of these agents across various medical specialties underscores the necessity of thoroughly assessing the clinical implications of their interaction. While the combination of prednisone and simvastatin may raise concerns regarding additive muscle toxicity, it is noteworthy that effective control of systemic inflammation through such therapeutic regimens can mitigate the risk of cardiovascular complications and osteoporosis, potentially reducing the need for additional pharmacological interventions. Interestingly, simvastatin may offer glucocorticoid-sparing effects. Studies have demonstrated that statins can decrease the number of tender joints, erythrocyte sedimentation rate, and C-reactive protein levels in RA patients, suggesting an anti-inflammatory benefit that could complement corticosteroid therapy^20,21^.

The high proportion of patients exposed to serious drug interactions presently verified highlights the imperative for routine clinical surveillance. Such interactions can lead to adverse outcomes, particularly among elderly individuals with multiple comorbidities, necessitating vigilant monitoring and individualized therapeutic strategies. The identification of clinically relevant drug-to-drug interactions allows early pharmaceutical interventions, reducing avoidable hospitalizations and complications in polymedicated patients.

This study has limitations such as its retrospective design, which restricted the collection of some additional information in the medical records. Furthermore, some interactions identified and classified as serious by the calculator should be carefully monitored by the attending physician, as the intended therapeutic use of these medications may inherently involve health risks. It is also worth noting that the MedScape^®^ platform did not include some medications, such as dipyrone, a commonly used analgesic in Brazil. Nonetheless, the calculator is user-friendly and free, and, in our opinion, provides valuable information for the management of patients with RA.

Our findings highlight the clinical relevance of polypharmacy in RA management, as the combination of immunosuppressants and medications for comorbidities significantly increases the risk of adverse events, hospitalizations, and treatment complexity. Furthermore, it guides healthcare professionals toward safer choices with less potential for harm, enabling individualized therapeutic decisions based on the patient's profile.

In conclusion, our findings showed that polypharmacy is prevalent in RA patients, particularly among those with a high number of comorbidities and advanced age. The study also showed that the MedScape^®^ platform successfully identified a substantial number of interactions, some of which were considered serious. Therefore, it is crucial to thoroughly examine these interactions, taking into account the risks and benefits.

## Data Availability

The datasets generated and/or analyzed during the current study are available from the corresponding author upon reasonable request.
